# A Pilot Study of the Nutrient Composition Diversity Index in a Sample of Healthy United States Adults Shows Positive Associations with Adherence to the Dietary Guidelines for Americans and Micronutrient Adequacy

**DOI:** 10.1016/j.cdnut.2025.107576

**Published:** 2025-10-14

**Authors:** Zachary P Gersten, Stephanie MG Wilson, Jules A Larke, Danielle G Lemay, Bess L Caswell

**Affiliations:** 1 Western Human Nutrition Research Center, Agricultural Research Service, United States Department of Agriculture, Washington, DC, USA; 2 Texas A&M AgriLife Research, Institute for Advancing Health Through Agriculture, College Station, TX, USA; 3 Department of Nutrition, University of California, Davis, Davis, CA, USA

**Keywords:** nutrient diversity, dietary diversity, nutrient intake, dietary quality, food dendrogram

## Abstract

**Background:**

Dietary diversity is an important component of dietary quality. The Nutrient Composition Diversity Index (NCDI) is a dietary diversity metric that quantifies dissimilarity among foods in a diet based on nutrient content. Research has yet to assess NCDI using an extensive food composition database and repeated 24-h dietary intake data or to test its associations with other aspects of dietary quality among United States adults.

**Objectives:**

This study aimed to describe NCDI constructed from dietary data collected over 2 quantitative 24-h recalls in a sample of healthy United States adults and to assess its relationships with adherence to the Dietary Guidelines for Americans and micronutrient intake adequacy.

**Methods:**

We analyzed cross-sectional dietary data from 377 participants (strata balanced for sex, age, and body mass index). Individual NCDI was scored using a dendrogram of 5628 foods and 30 nutrients from USDA food composition tables. We calculated usual NCDI to account for within-person dietary variability. Mean-standardized usual NCDI was compared with the 2015 Healthy Eating Index (HEI)-2015, HEI-2015 dietary components, and mean probability of adequacy for 17 vitamins and minerals in linear regression models, and with nutrient intakes using Pearson correlations.

**Results:**

Mean usual NCDI for the sample was 2.1% (standard deviation: 0.60), with an observed range of 0.81% to 4.0%; 70% of participants scored in the same or adjacent quintile of individual NCDI for their 2 recalls. NCDI was positively associated with HEI-2015 and with several HEI component scores while adjusting for total HEI-2015 (*P* < 0.0001). NCDI was also positively associated with nutrient intakes and with mean probability of adequacy in bivariate and energy-adjusted models (*P* < 0.0001).

**Conclusion:**

NCDI measures the dissimilarity dimension of dietary diversity and shows expected day-to-day variability and associations with adherence to Dietary Guidelines for Americans and micronutrient intake adequacy.

## Introduction

Dietary diversity has long been recognized as an important component of dietary quality [1]. Eating a variety of foods increases coverage to provide adequate intake of essential nutrients as well as dietary bioactive components that are not yet well-characterized [2,3]. For this reason, diversity of food intake is incorporated into the Dietary Guidelines for Americans and other food-based dietary guidelines as part of a healthy dietary pattern [1]. In addition to supporting dietary adequacy, dietary diversity itself may be an important exposure for some research, such as the study of links between diet, the gut microbiome, and immune function.

Concepts and methods from the field of ecology offer approaches to advance dietary diversity metrics. Reviews by Hanley-Cook et al. [4] and Bolo et al. [5] apply the concepts of evenness, richness, and dissimilarity used for biodiversity to dietary diversity and highlight the need for novel approaches to examine these specific facets of diet. Recent studies have shown that food species or flavonoid category richness are inversely associated with mortality in European and UK populations and that measures of food species richness and evenness are associated with lower mortality rates in European countries and with micronutrient intake adequacy in low-income countries [6,7].

In this study, we assess the dissimilarity dimension, the least explored by existing dietary metrics [5], by capturing the degree of difference in nutrient composition among foods that would provide a range of nutrients or other components. Previous researchers have drawn on the ecological concept of functional diversity to quantify the dissimilarity aspect of dietary diversity. Functional diversity defines the variety of life by ecosystem functions, rather than species, and is an indicator of ecosystem health and resilience [[8], [9], [10]]. The analogous nutrition concept—nutritional functional diversity—considers provision of nutrients as the functions of diet and expresses dietary diversity based on the dissimilarity of nutrient content among foods [2,[11], [12], [13]]. For clarity and to distinguish from the agricultural diversity metric, we will refer to this concept as nutrient composition diversity when applied to dietary intakes.

Although a number of studies have used the nutritional functional diversity approach as a measure of food production or household food purchase diversity [11,12,[14], [15], [16], [17], [18], [19]], we could locate only 2 that have assessed nutrient composition diversity of individual dietary intakes. Lachat et al. [20] and Di Maso et al. [21] used data from Italy and 7 low- and middle-income countries and found consistent positive associations with micronutrient intake adequacy and healthy dietary patterns. These studies used food composition databases containing 70 to ∼240 food items and either one 24-h dietary recall [20] or food frequency questionnaire data [21].

To our knowledge, how this diversity quantification method performs among United States adults, using complex food composition databases containing thousands of items, or with repeated 24-h dietary intake data, has not been demonstrated. Therefore, in this study, we piloted the Nutrient Composition Diversity Index (NCDI) using the USDA FoodData Central databases [22] and tested its performance using repeated 24-h dietary recalls collected in a sample of healthy United States adults. We hypothesized that NCDI would show positive associations with dietary quality as defined by the Dietary Guidelines for Americans, with nutrient intakes, and with overall micronutrient intake adequacy. The objectives of this study were as follows: *1*) to describe the distribution and day-to-day variability of NCDI constructed from dietary data collected over two 24-h recalls in sample of healthy United States adults; *2*) assess the associations of NCDI with dietary quality using the Healthy Eating Index (HEI)-2015 and its component scores; and *3*) assess the associations of NCDI with vitamin and mineral intakes and the mean probability of adequacy (MPA) for 17 vitamins and minerals.

## Methods

### Study design and data collection

This cross-sectional study analyzed repeated 24-h dietary recall data collected from 377 participants who were recruited in the Nutritional Phenotyping Study conducted at the USDA Western Human Nutrition Research Center [23]. The purpose of the study was to assess metabolic variability among healthy adults in balanced strata defined by sex, age, and BMI. The 3 age categories were defined by 16-y increments (18–33 y, 34–49 y, and 50–65 y); the 3 BMI categories matched the WHO classifications (normal: 18.50–24.99 kg/m^2^, overweight: 25.00–29.99 kg/m^2^, and obese: 30.00–39.99 kg/m^2^). The study was approved by the University of California, Davis Institutional Review Board, and registered at Clinicaltrials.gov (NCT02367287).

Study recruitment and enrollment occurred from May 2015 to July 2019. Participants were generally healthy adults who were living in the Davis, CA area. They were excluded if they were pregnant or lactating, had an egg allergy, underwent surgery in the previous 16 wk, received antibiotic therapy, were hospitalized in the past 4 wk, or were taking daily medication for a diagnosed chronic disease. Sociodemographic data, including age and sex, were collected using an electronic questionnaire through REDCap [24]. Height and weight for calculation of BMI were collected using a wall-mounted stadiometer and electronic scale (Scale-tronix 6002 or Tanita BWB-627A), respectively. Detailed information on study procedures is published in the protocol [23].

Dietary intake was assessed using the ASA24 automated self-administered 24-h recall from the National Cancer Institute (NCI) of the NIH [25]. Participants completed a training session with research personnel on how to use ASA24 during an initial study visit. Then, they completed ≤3 self-administered 24-h dietary recalls that were prompted on random days (2 weekdays and 1 weekend day) over a period of 10–14 d. Completed dietary recalls were cleaned for data quality based on ASA24 cleaning guidelines [26]. For this study, we analyzed the first 2 complete recalls for each participant due to ∼10% attrition for the third recall (*n* = 35). We used the NCI simple scoring algorithm to calculate total HEI-2015 score and the 13 HEI component scores over 2 dietary recalls [[27], [28], [29]].

### Usual nutrient intakes, PA, and MPA

To estimate usual nutrient intakes, we applied a Box-Cox transformation to the observed nutrient intake without supplements from each participant’s two 24-h dietary recalls, then estimated the best linear unbiased predictor (BLUP) of each participant’s usual nutrient intake in the transformed scale, following the measurement error approach described by Joseph and Carriquiry [30]. The BLUP of usual intake for individual *i* ($\tilde{y_{i}}$) is calculated using the following equation:

(1) $$\tilde{y_{i}}=\bar{Y\;}+\sqrt{\frac{\sigma_{yy}}{\sigma_{yy}+\sigma_{ww}/\;n_{i}}}\;\left(\bar{Y_{i}}-\bar{Y}\right)$$

in which $\bar{Y\;}$ is the mean of observed intakes over all recalls, $\sigma_{yy}$ is the between-person intake variance, $\sigma_{ww}$ is the within-person intake variance, $n_{i}$ is the number of recalls completed by individual *i*, and $\bar{Y_{i}}$ is the mean of observed recalls completed by individual *i*.

To calculate the probability of adequacy (PA) of 17 vitamins and minerals with dietary reference intakes [31], we compared each participant’s BLUP of usual nutrient intake to the Box-Cox–transformed age- and sex-specific requirement distribution. For each nutrient, the requirement distributions and nutrient intake data were Box-Cox transformed using the same value for λ. The PA for each micronutrient was defined as the percentage of the requirement distribution below the BLUP of usual intake [30,32]. We calculated the PA for the following vitamins and minerals: calcium, iron, magnesium, phosphorus, zinc, copper, selenium, vitamin A, thiamin, riboflavin, niacin, vitamin B-6, folate, vitamin B-12, vitamin C, vitamin D, and vitamin E. As a summary indicator of dietary quality, we assessed the MPA for each participant by averaging the PAs for the 17 vitamins and minerals [30].

### Nutrient Composition Diversity Index

**FIGURE 1:**
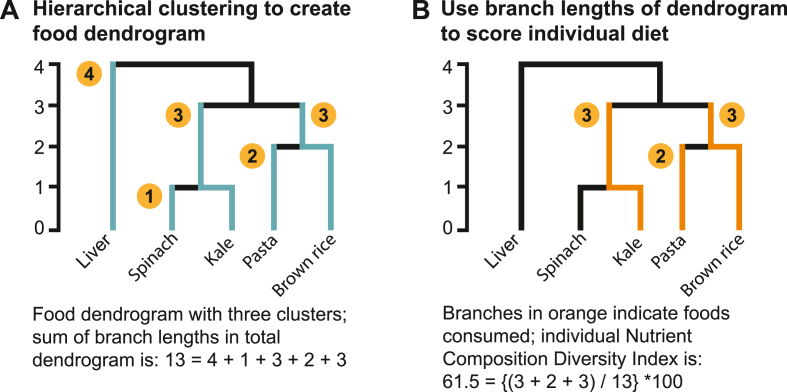
Calculation of the Nutrient Composition Diversity Index (NCDI) based on a food dendrogram. (A) Simplified example of a dendrogram organizing foods into clusters according to similarity of nutrient composition. The total nutrient composition diversity among foods in a food composition table is calculated as the sum of all branch lengths in the dendrogram. (B) Vertical branch lengths connecting foods consumed by an individual. To calculate the individual NCDI, these are summed and expressed as a percent of the sum of all branch lengths in the full dendrogram.

There are 4 steps to calculating the NCDI: *1*) create a nutrient trait matrix, similar to a food composition table, in which each row is a food item or ingredient, and each column contains a value for its nutrient content; *2*) calculate distances for all pairs of food items or ingredients in the nutrient trait matrix (Equation *1*); *3*) use hierarchical clustering to arrange food items and ingredients in a dendrogram and calculate the total vertical branch lengths (Figure 1A); and *4*) use the dendrogram to score individual diets by summing the vertical branch lengths to the common node (Figure 1B). The NCDI measures the diversity of foods in a diet based on the dissimilarity of nutrient contents.

To create the nutrient trait matrix, we adapted the USDA Food and Nutrient Database for Dietary Studies [33]. To reduce the number of food items in the nutrient trait matrix and facilitate future connection to food composition databases containing only single-food items, we disaggregated mixed food items to their ingredients using the recipe information provided in the USDA National Nutrient Database for standard reference (SR). We adapted existing code to carry out this process [34,35]. We removed ingredients without nutrient values, as these were generally additives or emulsifiers. During data collection, the NCI released a new version of ASA24 that shifted its food list from Food and Nutrient Database for Dietary Studies (FNDDS) 4.1 and SR 22 to FNDDS 2011–2012 and SR 23 [[36], [37], [38], [39]]. To ensure comparability of NCDI across ASA24 versions, we combined the 2 FNDDS and SR databases to create 1 harmonized nutrient trait matrix. Our final nutrient trait matrix contained 5628 single-food items or ingredients and their contents of energy and 29 nutrients with complete data. The macronutrients were protein, carbohydrates, total fat, dietary fiber, cholesterol, linoleic acid (18:2n–6) and α-linolenic acid (18:3n–3). The vitamins were vitamin A, α-carotene, β-carotene, thiamin, riboflavin, niacin, vitamin B-6, folate, vitamin B-12, vitamin C, vitamin D, vitamin E, and vitamin K. The minerals were calcium, iron, magnesium, phosphorus, potassium, sodium, zinc, copper, and selenium. We mean-standardized nutrient values to ensure their comparability regardless of measurement units [40].

In the second step, we calculated pairwise Euclidean distances for each pair of food items in the nutrient trait matrix (Equation *2*). This pairwise difference summarizes the dissimilarity of 2 food items or ingredients based on the number of nutrients with different values, as well as the size of those nutrient value differences.

(2) $$D_{i,j}=\sqrt{{\left(i_{protein}-j_{protein}\right)}^{2}+\ldots +{\left(i_{vitA}-j_{vitA}\right)}^{2}\;}$$

The third step is to apply a hierarchical clustering algorithm to the square matrix of Euclidean distances to identify clusters of food items or ingredients and produce a food dendrogram (Figure 1A). We used the unweighted pair group method with arithmetic mean algorithm. The distances between clusters and leaves (food groups and food items or ingredients, respectively) are considered the vertical branch lengths of the food dendrogram. The sum of all vertical branch lengths in the dendrogram represents the total nutrient composition diversity among food items and ingredients included in the nutrient trait matrix.

In the fourth and final step, we scored individual NCDI by summing the branch lengths from all recalled food items or ingredients to the common node, that is, where all branches meet, then expressed that as a percentage of the sum of branch lengths in the total dendrogram (Figure 1B). Similar to the nutrient trait matrix, we used the USDA SR recipe information to disaggregate the mixed food items reported in the dietary recalls to their ingredients and removed ingredients without nutrient values. NCDI is then calculated using binary variables indicating the food items or ingredients reported in each dietary recall. Because NCDI is expressed as a percentage of the total dendrogram branch lengths, the theoretical range is 0 to 100. However, because an individual’s daily diet will only cover a small fraction of the comprehensive foods list in the nutrient trait matrix, the practical range of NCDI is much lower. We used the vegan package (version 2.6-8) in R (4.4.1; R Foundation) to calculate the raw and total NCDI. We estimated untransformed, usual NCDI by calculating BLUPs as described earlier for usual nutrient intake.

### Statistical analyses

Data cleaning, descriptive statistics, calculating the BLUPs of usual NCDI, calculating the PAs and MPA, and the linear regressions were carried out using StataSE 18 (StataCorp; 2024) and SAS 9.4 (SAS Institute).

We used the mean (SD) and frequency statistics to describe the distributions of participant characteristics, PA, and MPA. We also assessed the proportions of the sample with adequate intake of each of the 17 vitamins and minerals. We used analysis of variance for comparisons of usual NCDI, HEI-2015 scores, and MPA by sex, age categories, and BMI categories.

To assess the day-to-day variability of the NCDI, we analyzed classification agreement by comparing quintiles of the NCDI for the 2 dietary recalls. We also visually assessed the distributions of the NCDI for the 2 dietary recalls, as well as the BLUP of usual NCDI. Finally, we fit a linear regression model to assess the relationship of standardized usual NCDI by standardized BLUP of usual energy intake.

We used simple linear regression models to estimate the bivariate relationship between HEI-2015 and mean-standardized usual NCDI. To test the association of NCDI with each of the 13 HEI components, we fit a series of linear regression models. In each, we regressed standardized usual NCDI on 1 HEI-2015 component score, controlling for the total HEI-2015 score calculated without that component.

We assessed Pearson product-moment correlations between usual NCDI and Box-Cox–transformed usual nutrient intakes and calculated 95% confidence intervals (CIs) using Fisher *z*-transformation. We fit 2 linear regression models of the association between standardized usual NCDI and Box-Cox–transformed MPA: the first being a bivariate model and the second with usual energy intake as a covariate. To illustrate effect sizes from these models in original scale, we calculated predicted values of MPA at −1, 0, and 1 SD NCDI, and at mean energy intake for the energy-adjusted model and back-transformed them.

## Results

The analytic sample included 377 participants who completed two 24-h dietary recalls and had complete sociodemographic data (Supplemental Figure 1). After disaggregating mixed foods, the mean (SD) number of distinct food items or ingredients reported per recall was 33 [12]. The mean (SD) of individual NCDI for both dietary recalls was 2.1% (0.84), with a range of 0.072% to 4.6%; mean usual NCDI was 2.1% (0.60) (Table 1), with a range of 0.81% to 4.0%. There was no statistical difference in usual NCDI by age categories or sex, but it tended to be lower in higher BMI categories. The mean HEI-2015 score was 61 [13]. Older age categories tended to have higher HEI-2015 scores, whereas higher BMI categories tended to have lower HEI-2015 scores. There was no statistical difference in HEI-2015 by sex. Mean MPA was 77% [17]. There was no statistical difference in MPA by age and BMI categories; mean MPA was higher in males than that in females.

**TABLE 1:** Usual NCDI, HEI-2015, and MPA for the sample and by age, sex, and BMI categories, among healthy adult participants in the USDA Nutritional Phenotyping Study, 2015–2019.

	% (*n*)	Mean (SD) dietary score		
		NCDI (%)^1^	HEI-2015 (0–100 point score)^2^	MPA (%)
Total sample	100 (377)	2.1 (0.60)	61 (13)	77 (17)
Age categories (y)				
<18–33.99	35 (132)	2.1 (0.66)	59 (13)	75 (19)
34–54.99	33 (126)	2.1 (0.57)	60 (12)	79 (14)
55 to >65	32 (119)	2.2 (0.57)	65 (13)	76 (17)
*P*^3^		0.567	<0.001	0.215
Sex				
Male	46 (172)	2.1 (0.58)	61 (14)	80 (15)
Female	54 (205)	2.1 (0.62)	62 (13)	74 (17)
*P*^3^		0.661	0.579	<0.001
BMI categories				
18.5–24.99	37 (140)	2.3 (0.61)	65 (13)	78 (17)
25–29.99	36 (135)	2.1 (0.55)	62 (12)	77 (17)
30–39.99	27 (102)	1.9 (0.55)	56 (12)	75 (16)
*P*^4^		<0.001	<0.001	0.291

Abbreviations: BMI, body mass index; BLUP, best linear unbiased predictor; HEI, Healthy Eating Index; MPA, mean probability of adequacy; NCDI, Nutrient Composition Diversity Index; USDA, United States Department of Agriculture.

1 Usual NCDI as the BLUP calculated from 2 dietary recalls.

2 HEI-2015 scores calculated using the simple scoring algorithm over 2 dietary recalls.

3 MPA calculated as the mean of probabilities of adequacy for 17 vitamins and minerals.

4 *P* values are for analysis of variance for comparisons of continuous variables.

**FIGURE 2:**
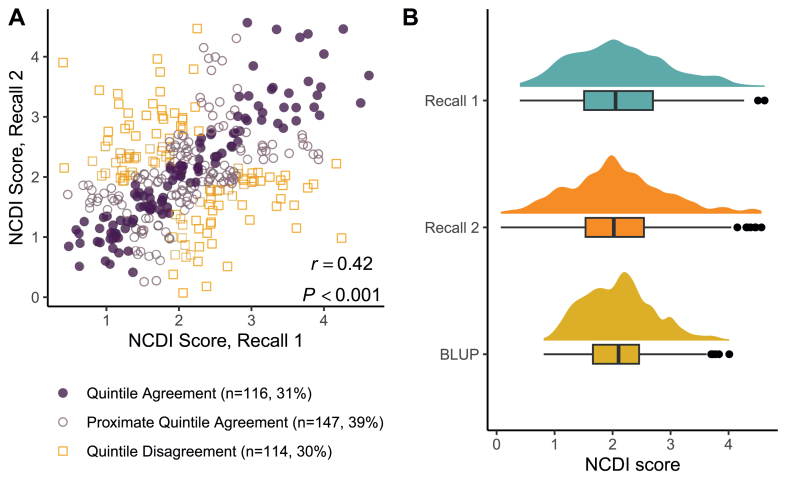
Day-to-day variability in the Nutrient Composition Diversity Index (NCDI) based on two 24-h dietary recalls collected among healthy adult participants in the USDA Nutritional Phenotyping Study, 2015–2019 (*n* = 377). (A) Scatterplot describing classification agreement between the quintiles of individual NCDI by recall. Quintile agreement is when quintiles of individual NCDI scores match; proximate agreement is for matches within 1-quintile difference (e.g., quintile 1 and quintile 2). (B) Distributions of the individual NCDI from the first and second recalls and the best linear unbiased predictor (BLUP) of usual NCDI.

The distributions of NCDI values were similar for the 2 dietary recalls, although a comparison of NCDI score and quintile classification showed day-to-day individual variability (Figure 2); 70% of participants were in the same or proximate quintile of individual NCDI for their 2 dietary recalls. A 1-SD increase in Box-Cox–transformed usual energy intake was associated with a 0.22-SD increase in usual NCDI (95% CI: 0.12, 0.32; *P* < 0.001).

A 1-point increase in HEI-2015 was associated with a 0.028-SD increase in usual NCDI (Table 2). Five HEI-2015 components showed statistically significant associations with standardized usual NCDI while controlling for HEI-2015 score without that component. One-point increases in individual dietary component scores were associated with 0.05- to 0.21-SD increases in usual NCDI for total vegetables, greens and beans, seafood and plant proteins, fatty acid ratio, and added sugars.

**TABLE 2:** Linear regressions of mean-standardized usual NCDI^1^ on HEI-2015 score and each HEI-2015 dietary component score among healthy adult participants in the USDA Nutritional Phenotyping Study, 2015–2019 (*n* = 377).

	β	*P*	95% CI
HEI-2015 score^2^	0.028	<0.001	0.021, 0.035
HEI-2015 dietary component scores^3^			
Adequacy			
Total vegetables^4^	0.205	<0.001	0.132, 0.279
Greens and beans^4^	0.173	<0.001	0.124, 0.221
Total fruit^4^	−0.017	0.5	−0.072, 0.038
Whole fruit^4^	0.006	0.8	−0.044, 0.056
Whole grains^5^	0.008	0.6	−0.024, 0.04
Dairy^5^	−0.015	0.4	−0.048, 0.019
Total protein foods^4^	0.096	0.1	−0.025, 0.218
Seafood and plant proteins^4^	0.147	<0.001	0.09, 0.203
Fatty acid ratio^5^	0.046	0.002	0.017, 0.076
Moderation			
Sodium^5^	−0.025	0.09	−0.055, 0.004
Refined grains^5^	0.005	0.7	−0.025, 0.036
Saturated fats^5^	0.016	0.3	−0.014, 0.045
Added sugars^5^	0.096	0.0001	0.049, 0.142

Abbreviations: BLUP, best linear unbiased predictor; CI, confidence interval; HEI-2015, Healthy Eating Index 2015; MPA, mean probability of adequacy; NCDI, Nutrient Composition Diversity Index; USDA, United States Department of Agriculture.

1 Usual NCDI as the BLUP assessed from 2 dietary recalls and mean standardized.

2 Unadjusted linear regression of mean-standardized usual NCDI on HEI-2015 total score (maximum score of 100) calculated using the simple scoring algorithm over 2 dietary recalls.

3 Linear regression of mean-standardized usual NCDI by each HEI-2015 component score, adjusted for total HEI-2015 score calculated without the individual component score included in the model.

4 Maximum dietary component score is 5.

5 Maximum dietary component score is 10.

Usual intakes of all nutrients were positively correlated with usual NCDI, except for cholesterol (Table 3). The magnitudes of the correlation coefficients were highest for vitamin K, vitamin A, β-carotene, α-carotene, vitamin C, vitamin E, magnesium, copper, and dietary fiber. The percentage of variation (*r*^*2*^) in usual nutrient intakes explained by usual NCDI ranged from 1.4% for niacin to 25% for vitamin K. Standardized usual NCDI was positively associated with Box-Cox–transformed MPA in both the bivariate (β: 0.026; 95% CI: 0.020, 0.032; *P* < 0.001) and energy-adjusted models (β: 0.017; 95% CI: 0.012, 0.022; *P* < 0.001). Back-transformed to original scale, the predicted values of MPA at −1, 0, and 1 SD usual NCDI were 76%, 81%, and 85%, respectively, showing a ∼5 percentage point change in MPA per SD increase in NCDI within the −1 to 1 SD range of the NCDI distribution. From the energy-adjusted model, at mean energy intake, back-transformed MPA predicted at −1, 0, and 1 SD NCDI was 78%, 81%, and 84%, respectively.

**TABLE 3:** Pearson correlations between the usual^1^ intakes of 30 macronutrients, vitamins, and minerals and usual NCDI among healthy adult participants in the USDA Nutritional Phenotyping Study, 2015–2019 (*n* = 377).

	ρ	*P*	95% CI
Macronutrients			
Total energy	0.22	<0.001	0.12, 0.32
Protein	0.17	0.001	0.07, 0.27
Total fat	0.20	<0.001	0.10, 0.29
Carbohydrates	0.22	<0.001	0.12, 0.31
Dietary fiber	0.44	<0.001	0.35, 0.52
Cholesterol	0.06	0.283	−0.05, 0.16
Linoleic acid (PUFA 18:2)	0.31	<0.001	0.22, 0.4
α-Linolenic acid (PUFA 18:3)	0.41	<0.001	0.32, 0.49
Vitamins			
Vitamin A (as retinol activity equivalents)^2^	0.41	<0.001	0.32, 0.49
β-Carotene	0.49	<0.001	0.41, 0.57
α-Carotene	0.45	<0.001	0.37, 0.53
Thiamin^2^	0.22	<0.001	0.13, 0.32
Riboflavin^2^	0.19	<0.001	0.09, 0.28
Niacin^2^	0.12	0.021	0.02, 0.22
Vitamin B-6^2^	0.26	<0.001	0.17, 0.35
Folate^2^	0.34	<0.001	0.24, 0.42
Vitamin B-12^2^	0.14	0.006	0.04, 0.24
Vitamin C^2^	0.38	<0.001	0.29, 0.47
Vitamin D^2^	0.15	0.005	0.04, 0.24
Vitamin E^2^	0.37	<0.001	0.28, 0.45
Vitamin K	0.50	<0.001	0.42, 0.57
Minerals			
Calcium^2^	0.18	<0.001	0.08, 0.28
Iron^2^	0.28	<0.001	0.19, 0.37
Magnesium^2^	0.39	<0.001	0.30, 0.47
Phosphorus^2^	0.25	<0.001	0.16, 0.35
Potassium	0.36	<0.001	0.27, 0.45
Sodium	0.23	<0.001	0.13, 0.32
Zinc^2^	0.18	<0.001	0.08, 0.28
Copper^2^	0.47	<0.001	0.38, 0.54
Selenium^2^	0.23	<0.001	0.13, 0.32

CI, confidence interval; NCDI, Nutrient Composition Diversity Index; PUFA, polyunsaturated fatty acid; USDA, United States Department of Agriculture.

1 Usual nutrient intakes and usual NCDI are calculated as best linear unbiased predictors based on 2 dietary recalls.

2 Vitamins and minerals used for calculating the mean probability of adequacy.

## Discussion

We piloted NCDI using a comprehensive food composition database and dietary data collected over two 24-h recalls in a sample of healthy United States adults. Our results demonstrate the day-to-day variability of nutrient composition diversity and its consistent relationships with nutrient intake adequacy and adherence to the Dietary Guidelines for Americans.

In this study, NCDI ranged between 0.072% and 4.6%, and usual NCDI, adjusting for day-to-day variability, ranged from 0.81% to 4.0%. Because we used the extensive USDA food composition databases as the basis of our nutrient trait matrix, individual daily intake can plausibly cover only a small percentage of the total food dendrogram. In our sample, participants reported an average of 33 foods per recall from a matrix including 5628 foods. In other dietary studies that used much smaller nutrient trait matrices, the ranges of nutrient composition diversity were higher and wider. In a cross-sectional sample of adults in Italy, Di Maso et al. [21] reported scores ranging from 0.23 to 0.88 (expressed as a proportion) using a nutrient trait matrix of 70 foods from a food frequency questionnaire. Lachat et al. [20] used a nutrient trait matrix of 234 plant and animal species in 7 low- and middle-income countries; although no range was presented, the mean nutrient diversity scores were comparable with that of Di Maso et al. [21].

We found within-individual daily variation in NCDI and that 30% of the sample exhibited a day-to-day shift between nonproximate quintiles. Within-person variability in nutrient diversity was heretofore unestablished, as previous studies quantifying nutrient composition diversity used either food frequency questionnaires capturing usual food intakes [21] or a single 24-h recall per participant when calculating the metric [20]. Our finding of day-to-day variability confirms the expected sensitivity of the NCDI metric to variation in foods consumed and supports use of modeling approaches to estimate usual NCDI when long-term average nutrient composition diversity is the exposure of interest [41].

NCDI showed positive associations with HEI-2015, nutrient intakes, and MPA. NCDI was also independently associated with several HEI component scores while controlling for the total HEI-2015 score. NCDI showed the strongest positive associations with HEI component food groups that contain greater diversity. For example, total vegetables includes a wide variety of species, varieties and parts of the plant consumed. The positive association between standardized usual NCDI and the added sugars HEI-2015 moderation component may reflect trade-offs in consumption of micronutrient-diverse, minimally processed foods and highly processed foods, which are generally composed of the same refined ingredients and commonly have high fat, salt, and/or sugar content [42].

We found that within 1 SD of the mean usual NCDI, MPA increases by ∼5 percentage points per 1-SD increase in NCDI, suggesting that a diet of foods with more dissimilar nutrient profiles is more likely to provide adequate intakes of a range of micronutrients than a diet of foods with more similar nutrient profiles. This effect size is similar to the 3 to 4 percentage point increase in MPA per additional food group consumed among United States adolescents reported by Jenkins et al. [43]. As expected, we found a slightly attenuated association between NCDI and MPA when adjusting for usual energy intake, which matches findings in a systematic review of studies of dietary diversity indicators and their associations with dietary adequacy [1]. The relationship between nutrient composition diversity and adequacy of the diet is further supported by the positive correlations observed between NCDI and individual nutrient intakes.

Our findings of positive but weak associations with HEI-2015, nutrient intakes and MPA align with the concept that nutrient composition diversity, adherence to dietary guidelines, and nutrient intakes are distinct but related facets of dietary quality. The findings in this analysis also align with previous research on associations between nutrient composition diversity and dietary quality in both high-income and low- and middle-income country contexts [20,21]. This emerging body of findings suggests that this approach is applicable in multiple contexts and with different types of dietary intake data, while addressing the concern that some metrics of dietary diversity have been negatively associated with dietary quality in high-income countries [1,5].

The data-driven approach to organizing the food dendrogram, which allows the same method to be applied to different study contexts, objectives, and food composition tables, is a strength of NCDI. Although our study examined 30 nutrients from the USDA FNDDS and SR, prior studies used a similar approach with different macro- and micronutrient composition tables. The method could also be applied to create food composition dissimilarity indices on other dietary components, such as polyphenols or glycans. An alternative approach is to calculate metrics from tree diagrams based on a predetermined food categorization scheme. Kable et al. [44] and Johnson et al. [45] have quantified dietary diversity using a phenetic tree created from the hierarchical food coding system in the FNDDS. This tree-based metric has been associated with gut microbiome composition, but, to our knowledge, has not been assessed relative to dietary quality, dietary adequacy, or nutritional status markers [44,45]. Unlike nutrient composition diversity, this dendrogram method cannot be applied to dietary data not coded with FNDDS or a similar hierarchical food code system, and the distance estimation is inconsistent with respect to dissimilarity of nutrient content.

Limitations of NCDI relate to trade-offs made to favor interpretability and applicability. The flexibility to change the foods or dietary components included in the metric presents the limitation that NCDI is only comparable between individuals or groups when it is calculated from the same dendrogram. Future studies comparing across populations would need to map dietary data to a single food composition table or append multiple tables into a single food trait matrix before compiling the dendrogram and calculating NCDI. We also included the substantial data processing step of disaggregating mixed dishes. However, disaggregation substantially reduces the number of items in the nutrient trait matrix and allows diets to be compared at the ingredient level. Evaluation at the ingredient level supports interpretation and linkage to other food composition databases that do not contain mixed dishes. More broadly, calculation of NCDI requires expertise in dietary data and statistical programming. It also requires a complete list of specific foods consumed, although portion size data are not needed. For some research contexts, these factors may be significant limitations in contrast to the data and calculation requirements for food or food group count metrics of dietary diversity.

Although our pilot use of NCDI to evaluate dietary diversity among United States adults generated promising findings, the results have limited generalizability. The USDA Nutritional Phenotyping Study, the source of data for this analysis, enrolled healthy people living in and near Davis, CA, in balanced age, sex, and BMI categories [23]. Thus, the sample is not representative of the overall United States population. An important next step in the evaluation of NCDI as a novel dietary diversity metric will be to test its use in other populations, including nationally representative samples.

Other directions for future research on nutrient composition diversity include exploring its determinants and relationships with health outcomes. In previous studies, application of a nutrient diversity metric to food production or purchase data has revealed connections to food production and access patterns, diet, or socioeconomic factors [11,12,14,16]. NCDI may be similarly applicable to questions of food access and diet in the United States. The phenetic tree-based diversity metric was shown to relate to gut microbiome composition, and other authors have suggested that novel dietary diversity metrics may be useful in studying the role of diet in immune function, aging processes, and chronic disease risk [[44], [45], [46], [47]]. In particular, the dissimilarity aspect of diet may be of particular relevance in studying health effects mediated by the gut microbiome. Accounting for the diversity of dietary substrates, such as the range of fiber types, may improve prediction of microbial responses to diet [48]. Diverse diets have also been linked to microbial diversity [49] and functional capacity [50]. Assessing nutrient dissimilarity in similar capacities may clarify whether microbial relationships are driven by nutrient composition diversity. The flexibility of NCDI to express the compositional diversity of different sets of nutrients and bioactive dietary components may add to its usefulness in these areas of research.

In conclusion, we have shown that NCDI can be calculated from extensive United States dietary reference databases to express the dissimilarity of nutrient composition among foods in United States adult diets reported by 24-h recall. In this sample, NCDI showed the expected associations with measures of dietary quality and adequacy. Future research should further test its generalizability in different populations, evaluate associations with determinants of diet and with health outcomes, and apply the NCDI method to different sets of nutrient traits.

## Author contributions

The authors’ responsibilities were as follows—ZPG, BLC: designed and conducted the research; ZPG: performed statistical analysis; SMGW, JAL: provided analysis code and supported analysis; SMGW: provided figures; DGL: conducted nutritional phenotyping study and provided data for this analysis; ZPG, BLC: wrote the manuscript; ZPG, BLC: had primary responsibility for the final content; and all authors: read and approved the final manuscript.

## Data availability

Data described in the manuscript and analytic code will be made available upon request pending application and approval by the USDA ARS WHNRC Nutritional Phenotyping Study Investigators. Requests should be sent to the corresponding author. Sample code for constructing the Nutrient Composition Diversity Index using 24-h recall data coded using the USDA Food and Nutrient Database for Dietary Studies and National Nutrient Database for Standard Reference will be made available on GitHub (https://github.com/BessCaswell-USDA/NCDI).

## Funding

This research was supported by the USDA Agricultural Research Service (ARS, Project No. 2032-10700-002-000-D) and the USDA ARS Postdoctoral Research Associate Program. The funder had no role in study design, data analysis, decision to publish, or preparation of the manuscript.

## Conflict of interest

The authors report no conflicts of interest.
