# Supplementary material for: A Pilot Study of the Nutrient Composition Diversity Index in a Sample of Healthy United States Adults Shows Positive Associations with Adherence to the Dietary Guidelines for Americans and Micronutrient Adequacy
Source: Curr Dev Nutr. 2025 Oct 14;9(11):107576. doi: 10.1016/j.cdnut.2025.107576 (PMC12670087; doi:10.1016/j.cdnut.2025.107576)

Supplementary Figure 1. STROBE diagram for analytic sample drawn from USDA Nutritional Phenotyping Study, 2015-2019


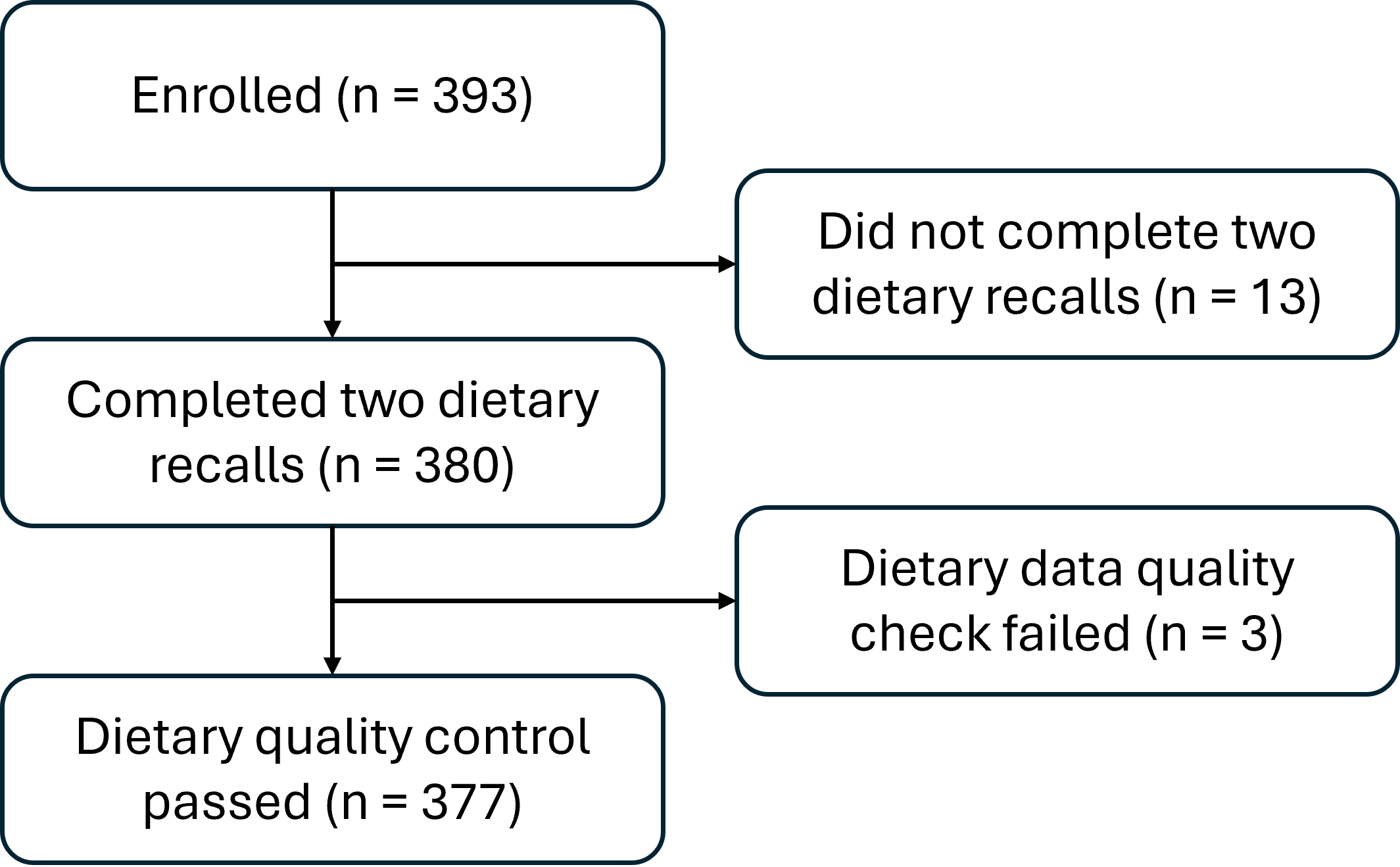

Supplement: Multimedia component 1 [file mmc1.docx]
